# Using guidelines to improve neonatal health in China and Vietnam: a qualitative study

**DOI:** 10.1186/s12913-016-1900-x

**Published:** 2016-11-11

**Authors:** Joanna Raven, Xiaoyun Liu, Dan Hu, Weiming Zhu, Dinh Thi Phuong Hoa, Le Minh Thi, Doan Thi Thuy Duong, Alvaro Alonso-Garbayo, Tim Martineau

**Affiliations:** 1 Department of International Public Health, Liverpool School of Tropical Medicine, Pembroke Place, Liverpool, L3 5QA UK; 2 China Centre for Health Development Studies, Peking University, Haidian, China; 3 Department of Reproductive Health, Hanoi school of Public Health, Hanoi, Vietnam

**Keywords:** Neonatal health, Guidelines, China, Vietnam, Behaviour change

## Abstract

**Background:**

Neonatal health (NH) remains a major problem in many countries. Children dying before 28 days often suffer from conditions that are preventable or treatable with proven, cost-effective interventions. The knowledge gaps are no longer about what should be done, but to understand why guidelines including these interventions are not followed. Using a behaviour change framework, this study explores neonatal health guidelines use and the role of management in supporting effective usage in two rural settings in China and Vietnam.

**Methods:**

Semi-structured interviews with policy makers, health care managers and providers (*n* = 49) and focus group discussions with women, husbands and grandmothers who had experienced maternal and NH care services within the last year (*n* = 7) were conducted. Data were analysed using the framework approach.

**Results:**

Guidelines are not readily available at county, township and village levels in the study sites in China, whereas, in Vietnam, guidelines are available, accepted and being used at facility level. Improvements in implementation could be made in both settings. Factors influencing guidelines use common to both settings included: lack of equipment and supplies; shortage of staff with NH care experience; and guidelines not in line with patient practices. Factors specific to China included: poor guidelines dissemination; and disagreement with guidelines. There was limited community engagement in NH services in China, whereas in Vietnam, community members were actively involved in decision making and provision of services. Managers have an important role in supporting NH guidelines use through: ensuring guidelines are available; allocating appropriate resources; supporting and monitoring staff in their use; and engaging with local communities to promote effective practices.

**Conclusions:**

Engaging managers to support implementation is crucial. Management systems that provide the necessary resources, competent staff, and monitoring, regulatory and incentive frameworks as well as community engagement are needed to promote adoption of guidelines. Further research on how best to strengthen local level management so that they tailor interventions to support guideline use to their specific context is needed. This will ensure that proven interventions to address NH problems are used, and that countries move closer to achieving the new Sustainable Development Goal 3 target.

**Electronic supplementary material:**

The online version of this article (doi:10.1186/s12913-016-1900-x) contains supplementary material, which is available to authorized users.

## Background

Although there has been progress globally in meeting the targets of Millennium Development Goal 4, neonatal mortality rates (NMR) have lagged behind [1] with 45 % of under five deaths globally occurring in the neonatal period, the majority within the first few days of life [2]. The ambitious and challenging target for neonatal mortality in Sustainable Development Goal 3 (SDG), is a reduction from the current 19 deaths to less than 12 deaths per 1000 live births [3]. The neonatal period is the most vulnerable time for a child’s survival and children that die before 28 days of life often suffer from diseases and conditions that are readily preventable or treatable with proven, cost-effective interventions.

Much research has been carried out to find solutions to the clinical aspects of neonatal health (NH). For example, we know that highly cost-effective interventions are feasible at facility and community levels such as early postnatal home visits to promote breastfeeding and clean cord care, skilled care at birth and kangaroo mother care [2, 4]. Between 40 and 70 % of neonatal deaths could be averted by full coverage of 16 feasible interventions [5]. These interventions are included in guidelines.

Guidelines are “systematically developed statements to assist practitioner and patient decisions about appropriate health care for specific clinical circumstances” [6]. Guidelines are developed on global best practice based on the best available evidence, which are then adapted to local conditions as necessary. The use of guidelines should lead to changed health worker behaviour, which will then lead to better health outcomes [7, 8] and the achievement of neonatal health target in Sustainable Development Goal 3.

There are many factors that influence the implementation of guidelines. These include: the quality of guidelines (i.e. relative advantage, compatibility with existing beliefs and values, complexity, and cost); the ability of the provider to try out the guidelines with comparative ease; the ability of the provider to observe practices that have incorporated the new guidelines; characteristics of the health care professional; characteristics of the practice setting such as incentives and regulations; patient factors such as individual demands or specific clinical problems [9]. Different types of health care providers may require different strategies to facilitate their adherence to guidelines [10].

In addition, communities are an important component of the health system and, as such, can take an important role in the successful implementation of guidelines. For example, increasing community awareness about the importance of appropriate NH care increases service demand which can have an impact on providers’ adoption of guidelines [11].

**Fig. 1:**
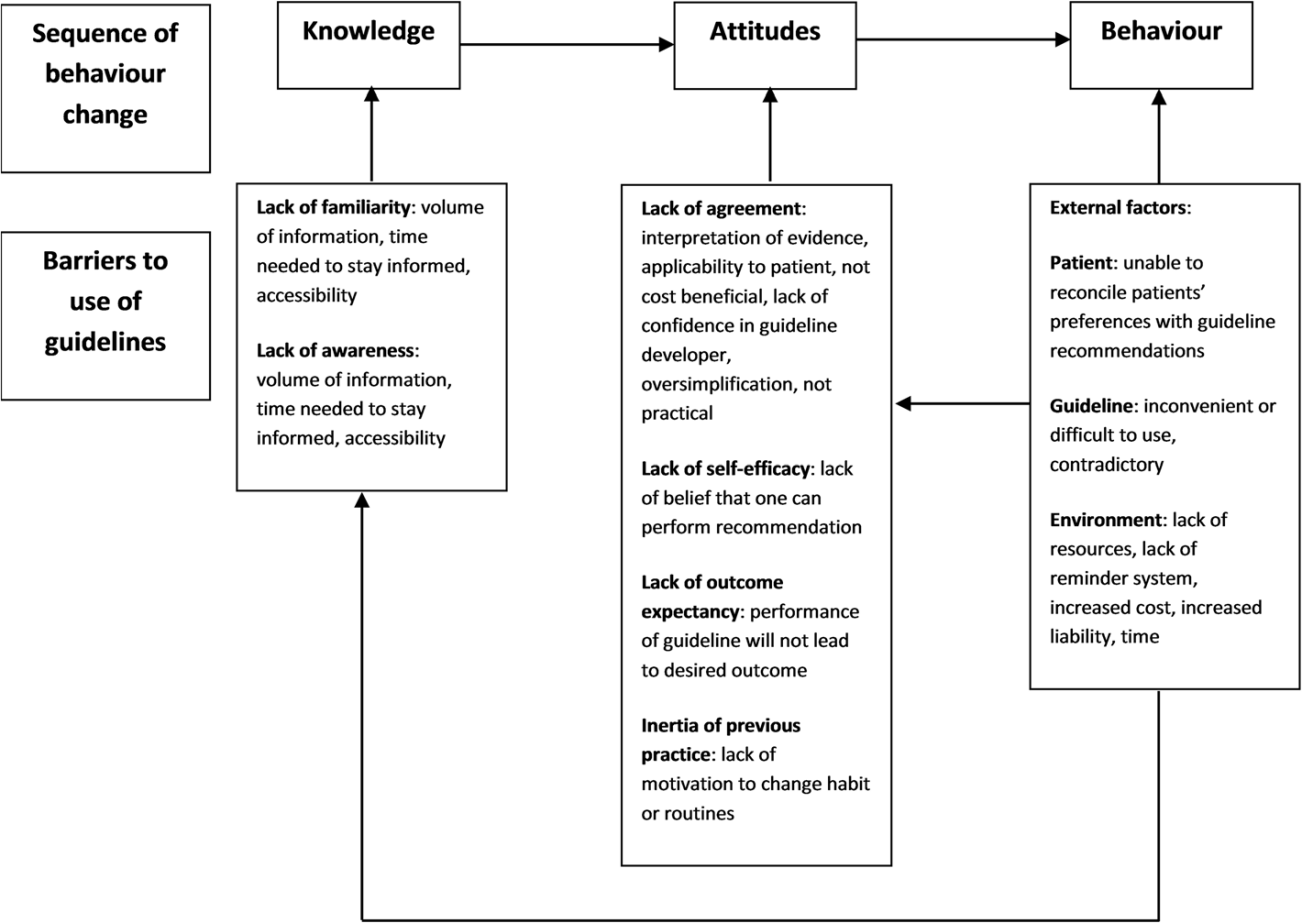
Barriers to guideline usage (adapted from Cabana et al. [6])

In their framework based on a behaviour change perspective, Cabana et al. identified three main areas where barriers may exist in relation to the providers: knowledge, attitudes and behaviour or practice [6] (Fig. 1).

Cabana et al.’s framework does not explicitly include the role of managers in implementation of guidelines. Managers can influence how guidelines are implemented and this depends to some extent on how much room for manoeuvre they have to make decisions such as resource allocation or addressing training needs. Room for manoeuvre or “decision space” is described by Bossert as the degree of autonomy with which managers make decisions according to their decentralized authority [12]. It is also important for them to monitor how guidelines are used, and their effect on health outcomes.

Despite impressive development and economic growth in both China and Vietnam, NH is still a major problem. In China, neonatal mortality accounts for 60 % of all childhood deaths [13] while in Vietnam, neonatal mortality comprises nearly three-quarters of all infant deaths [11]. Higher rates of neonatal mortality exist in poorer and remote rural areas of both countries. We also know that NH practice guidelines exist in China and Vietnam for use at national and local levels [14, 15]. However, a major problem is ineffective implementation of these guidelines in service delivery, which is reflected in, for example: low rates of postnatal care [16]; poor quality neonatal resuscitation [17, 18]; and low rates of early and exclusive breastfeeding [19, 20].

Since evidence about effectiveness of interventions to address NH problems is broadly available, the knowledge gaps are no longer about what should be done to improve NH outcomes. Rather, “implementation research” [21] is urgently needed to understand why guidelines are not followed, and what room for manoeuvre managers have to support implementation of NH practice guidelines effectively and to help health workers foresee and respond to problems in implementation. This paper presents findings from preliminary research to identify research gaps and questions surrounding the use of NH guidelines. The aim of this study was to explore the availability of guidelines in the two study sites, how they are used, the challenges in usage faced at different levels within the health system, the managers’ role in use of guidelines, and the community involvement in neonatal health care.

## Methods

This was a small-scale preliminary study to explore the use of NH guidelines in order to inform the design of a larger implementation research study.

### Research team

The Chinese research team collected and analysed the data from the Ningxia study. The team consisted of a medical doctor, a social scientist, and health service management researcher. The Vietnamese research team, consisting of medical doctors and social scientists carried out the data collection and analysis. The UK team supported the design, data collection and country and comparative analysis. The team consisted of a social scientist with midwifery experience, and health service management researchers.

### Study sites

The research was carried out in Ningxia Hui Autonomous Region, China, and Dak Nong Province in Tay Nguyen Region, Vietnam. These sites are amongst the poorest regions in each country, with below average national NH indicators, and the research teams have existing relationships with institutions in these regions. They were selected as potential significant impact on NH indicators could be achieved in the implementation study.

Ningxia is an autonomous region located in the North West part of China. It is a mountainous and desert like region, with difficulties in health care access. The total population in 2012 was 6.47 million of whom 33 % are Hui/Muslim. The NMR in Ningxia was 10.4 per 1000 live births in 2010 [22], whereas the national average lies at 8.3 per 1000 live births [23]. Maternal mortality is also higher in Ningxia, with a ratio of 27.5/100,000 live births in 2012 (national average is 24.5/100,000 live births) [24]. Within Ningxia, Xiji County was selected as the study site. Xiji is remote and rural, is designated as a “national poverty county”, and has a population of half million working mainly in agriculture.

Dak Nong province is one of the poorest of the five provinces in the Tay Nguyen region. It has a population of 550,000 of whom 16 % are considered poor [25]. 40 % of the population belong to 40 ethnic groups [26]. There are many challenges for ethnic minority people in accessing health care such as geographical distance, costs, transport and cultural barriers [27]. While the officially reported NMR for Tay Nguyen is 4.2 per 1,000 live births, a survey estimated it to be 15.3 per 1000 live births [28]. Within Dak Nong province, Dak Glong district and Dak R’Mang commune were selected as study sites. Dak Glong is a remote area and is one of the poorest districts in the province with 49 % of households being classified as poor [25].

### Study design

This was a small-scale preliminary study to explore the use of NH guidelines to inform the design of a larger study. Little is known about the use of NH guidelines in these settings, and therefore a qualitative research methodology was used to explore their use including any barriers and opportunities, amongst a variety of stakeholders. Semi-structured interviews and focus group discussions were employed. Data collection was carried out by the country research teams between October and December 2014.

### Semi-structured interviews with policy makers, managers and health workers

Semi structured interviews (SSI) were used as they ensured that key topics were covered in each interview, and allowed deeper exploration based on the participants’ responses. This facilitated comparison of findings across the two country contexts. Key informants who are involved in neonatal health policy making and implementing at national, provincial, and county/district levels, managers of neonatal health care services at county/district level, and health care workers who provide neonatal health care services were selected for interview. The sampling approach was purposive aiming to capture a range of views and perspectives by experiences, and location in the health system in order to enable “symbolic representation” [29]. A total of 49 interviews were conducted – Table 1 provides details of the selection in each country.

**Table 1:** Numbers and types of participants for semi-structured interviews

	Vietnam		China	
Level	Type of informant	No.	Type of informant	No.
National	Director Maternal and Child Health (MCH) Department, Ministry of Health UNICEF representative	2	Director National Centre for Disease Control and Prevention, MCH centre UNICEF representative Research expert on NH health	3
Province	Policy makers: Director Provincial Health Department Manager Provincial Reproductive Health Centre	2	Policy makers: Director Provincial Health Department MCH division Manager Provincial MCH Centre	2
	Senior health service professionals: 1 Medical Professional Division, Provincial Health Department 3 officers Provincial Reproductive Health Centre 2 health workers, Obstetrics department, Provincial Hospital 2 health worker, Paediatrics department, Provincial Hospital	8	Senior health service professionals: Director Paediatric Department, Provincial Hospital	1
District/County	1 vice director, district hospital 1 head, Obstetrics department 1 head, Paediatrics department 1 midwife, Obstetrics department 1 MCH manager, District Preventive Medicine Centre 1 officer, District MCH centre 1 midwife, MCH department	7	1 Director, County Health Bureau 1 MCH Manager 3 Managers, township hospitals 4 health workers, township hospitals 2 health workers, county hospital	11
Commune/village	1 head, Commune People’s Committee 1 head, Commune Health Centre 1 midwife, Commune Health Centre 1 village midwife 3 village health workers 2 representatives, Village Women’s Union	9	3 village doctors 1 representative, Women’s Union	4
Totals		28		21

Topic guides were developed by the research teams and covered areas such as neonatal health issues, availability of NH guidelines, use of guidelines and challenges, how neonatal health is monitored, degree of freedom of district or county level managers have to ensure the implementation of guidelines and authority to decide on use of resources to more successfully implement the guidelines (see Additional file 1). All interviews were recorded and took place in a private place acceptable to the interviewees.

### Focus group discussions with community members

A total of seven focus group discussions (FGDs), three in China and four in Vietnam, were conducted to explore mothers’ and family members’ perceptions and experiences of NH care services and views on ways to improve service delivery. Women, husbands and grandmothers who had experienced maternal and neonatal health care services within the last year were selected for the FGDs. The FGDs, separated by gender and included between five and nine participants, were facilitated using an open-ended topic guide in private places acceptable to the participants (see Additional file 1). The discussions were recorded.

We aimed to arrive at the sample size following the principle of saturation whereby interviews and discussions should continue until no new data are generated [29]. Saturation was achieved with regard to the key topics.

### Analysis of data

Recordings of the interviews and discussions were transcribed verbatim in the local language and checked for accuracy. These data were analysed in the local language by the Chinese and Vietnamese research teams using the framework approach which facilitates rigorous and transparent analysis [30]. Firstly, coding frameworks were developed using the topic guides, study objectives and themes emerging from the data, and so were slightly different for each country. Then the coding frameworks were applied to the transcripts, charts were developed for each theme, and these charts were used to describe the themes. The computer programmes MAXqda and NVIVO 8 were used to support the analysis. The findings were then compared across the two contexts through a process of joint meetings and discussion between Chinese, Vietnamese and UK researchers.

### Ethical considerations

Ethical approval was obtained from the Research Ethics Committee at Liverpool School of Tropical Medicine (14.041), the Ethics Committee for Biomedical Science in Peking University (IRB00001052-14063) and the Research Ethics Committee at Hanoi School of Public Health (285/2014/YTCC-HD3). Informed consent was obtained from each participant prior to starting and recording the interviews and discussions.

## Results

### Use of guidelines

In Vietnam, some aspects of the neonatal health guidelines included in the National Guidelines for Reproductive Health Care Services were reported by the respondents to be implemented at provincial, district and commune level. These included initiation of breast-feeding within 1 hour after normal delivery, new-born examinations, and safe referral of sick new-borns to the higher levels. However, it was reported that other neonatal health care was not implemented fully according to the guidelines, such as kangaroo mother care, cord care, diagnosis and treatment of sepsis, early initiation of breastfeeding following caesarean section, and care of pre-term babies.

In China, we found that guidelines were not routinely used to guide provision of NH care. Although key informants at national level claimed that these guidelines are well applied, interviews at provincial, county, township and village levels did not confirm this. Staff at provincial level hospitals use international guidelines which they retrieve from the internet. Health workers at county and township levels either follow their past experience or use textbooks.

### Factors affecting guideline use

**Fig. 2:**
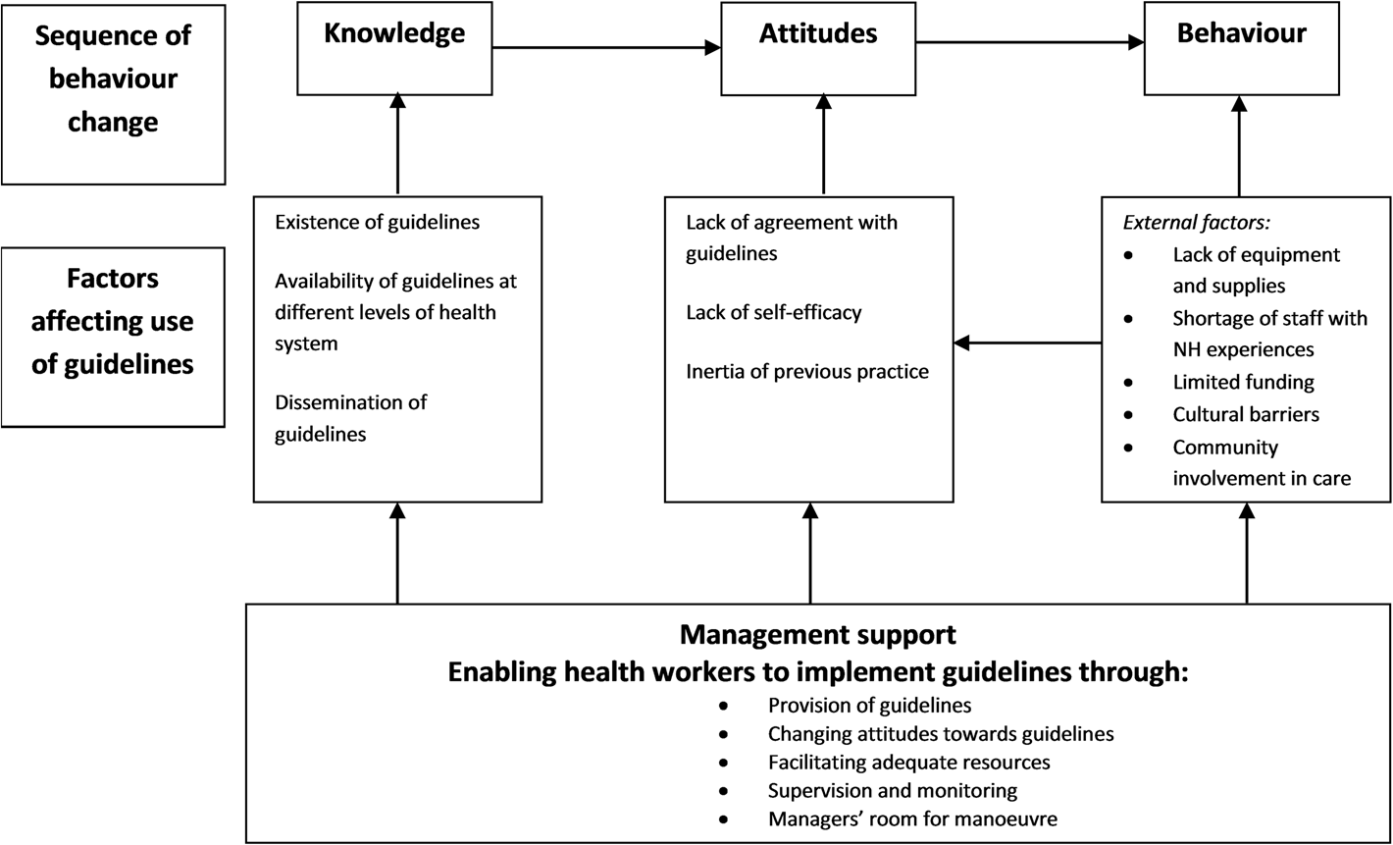
Factors influencing use of guidelines in China and Vietnam

The factors are described in four categories: knowledge of the guidelines, attitudes towards the guidelines, factors that influence the behaviour of the health workers and management support. Figure 2 is an adaptation of the Cabana model which summaries these factors [6].

#### Knowledge

##### Existence of guidelines

In 2009, the Vietnam Ministry of Health (MoH) issued the National Guidelines for Reproductive Health Care Services (NGRHS), including one chapter on NH care with 40 topics covering almost all new-born problems, to be used at both government and private health facilities. These guidelines were developed by national and international experts on NH from MoH, hospitals, WHO, UNICEF, Save the Children, and UNFPA.

In China, respondents reported that there are three types of guidelines for NH. There are clinical guidelines that provide detailed information on how to provide care. They are usually developed by professional associations (which have strong government links) which are published in academic journals, and include guidelines on neonatal resuscitation, management of premature babies, and breastfeeding. There are guidelines on delivery of services such as the frequency and content of postnatal visits. Third, there are textbooks, wall charts and newsletters.

##### Availability of guidelines at different levels of the health system

In Vietnam, the NGRHS were available at the provincial hospital, district hospital and commune health station. The guidelines were also available at the provincial health department, provincial centre of RHC, and district preventive health centre as they are used for monitoring and evaluation, health information statistics, and training and research purposes.

*“We received the reprinted version of the national guidelines from the Provincial Health Department in 2011. The guidelines were reprinted using provincial budget. All health facilities received the guidelines”* (Manager, provincial level, Vietnam).

In China, the clinical and service delivery guidelines are available on the MoH website. All township health centres and many village health clinics have internet access. However, they were not available in hard form within the health facilities at province, county, township and village level. There were wall charts explaining frequency and content of postnatal health care visits and neonatal resuscitation in the township health centres and village clinics. The health workers reported keeping a textbook at home that includes sections on neonatal health care.

##### Dissemination of guidelines

In Vietnam, there was a systematic approach to the dissemination of the guidelines, with each level of the health system being responsible for disseminating the guidelines to the health facilities, introducing the guidelines at regular meetings, and organizing training for managers and health workers. This training was conducted when the guidelines were first introduced and then every year in order to update staff on any changes in the guidelines. In China, neonatal health guidelines are distributed from the central MCH level to lower level MCH specialists. Training on the use of guidelines is provided by higher level institutions but this was reported to be ad hoc, did not adequately cover emergency treatment of some neonatal complications, and did not include opportunities for practicing and developing skills.

##### Management support: provision of guidelines

These findings indicate that in Vietnam, there appears to be clearer communication about the guidelines and a systematic approach to ensuring that staff at all levels have access to and are knowledgeable about the guidelines. In China, there are reported problems with communication of guidelines at the different levels within the health care system, resulting in the guidelines not being available at the lower level facilities.

#### Attitudes

##### Lack of agreement with guidelines

Some respondents reported that the China national guidelines are seen as being out of date, do not include important interventions and are not compatible with international guidelines. Health care providers at provincial level were reported to prefer to use international guidelines. A respondent explains:

*“Kangaroo mother care is the most cost-effective intervention to premature and low weight babies…However, kangaroo mother care is not mentioned in any of our guidelines”* (Key informant, national level, China).

##### Lack of self-efficacy

In Vietnam, a systematic approach to training health managers and health workers on NGRHS was reported. A training manual was developed and provincial level trainers were trained in conducting workshops for health workers at district hospitals and community health centres. Every year health workers from all health facilities receive training in the updated guidelines at the Provincial Reproductive Health Centre. Experts are sometimes invited from paediatric hospitals in Ho Chi Minh City to deliver the training. However, some respondents reported that the training focuses on theory, and there are no opportunities to practice skills on models or patients. Health workers do not feel confident to apply these skills in their work. Furthermore, not all health care providers receive the annual training as this would leave their facility understaffed.

*“The training in the province is in theory only. It’s hard to understand without a model. We have to invite experts from Ho Chi Minh City to our hospital to train our doctor, especially the complicated techniques”* (Manager, provincial level, Vietnam).

*“Our department has only one doctor, I could not attend the training*” (Health worker, district level, Vietnam).

In China, respondents reported that staff at township and village levels do not have the necessary skills to provide care according to the guidelines because their initial training of 3 years is not adequate, and they do not receive sufficient and effective in-service training to upgrade their skills. There are few health workers that specialize in NH care.

##### Inertia of previous practice

In China, there is evidence that health workers provide care according to habit and routine rather than use the guidelines. Respondents reported that health workers at county and township levels use textbooks that were published decades ago as their guidelines. These textbooks were followed during their training and they are accustomed to using these textbooks. Health workers tend to follow more senior staff, so if the head of department always uses a certain method in treating a disease, then the staff will follow this practice. Guidelines are only going to be followed if staff are willing and able to adopt them into their heavy and complex workload, as illustrated by this respondent:

*“Guidelines are only helpful to village doctors who are willing to learn more. Since the workload of village doctors is complex and large, plus their low motivation to do the work, some of them may not refer to guidelines. Therefore, even if the village doctors are equipped with guidelines, it still may not achieve expected effect”* (Key informant, provincial Level, China).

##### Management support: Changing attitudes

These findings suggest that the role of the manager should include identifying when it is necessary to change health workers’ attitudes about using guidelines and then facilitate this change. Their role is also to change attitudes towards the content of the guidelines. For example, in China the guidelines did not include kangaroo mother care but the manager saw this as an important intervention to support premature and small babies.

#### Behaviour

Behaviour of the health workers to implement the guidelines is influenced by the following external factors: equipment and supplies, staffing, funding, cultural practices and community involvement.

##### Lack of equipment and supplies

Lack of equipment and drugs hinders provision of neonatal health care services according to the guidelines, and this is particularly evident at the primary health system levels. For example, in Vietnam, health workers explained that lack of drugs such as surfactant (drug used with neonates with respiratory distress), and limited equipment such as delivery sets hampered their ability to provide delivery services as well as care for sick or premature babies. In China lack of equipment to conduct neonatal hearing test limits health workers’ capacity to follow the hearing screening guidelines.

*“We can take care of some premature babies. However, the lack of drugs such as surfactant prevents us from keeping premature babies in the hospital”* (Health worker, provincial level, Vietnam)*.*

*“As for equipment we have only one professional ultrasound Doppler, two delivery kits and two perineal suture kits. That's all! Tools are inadequate, heat lamps for babies are also not available in this hospital”* (Health worker, district level, Vietnam)*.*

##### Shortage of staff especially with NH expertise

Respondents identified two issues about staffing for neonatal health care in China and Vietnam. There is a general lack of staff in rural areas particularly at district/county, township/commune and village levels. The existing staff are therefore overloaded with other public health and clinical activities, and are unable to devote enough time to NH care. In addition, many staff also lack skills and experience in neonatal health care. For example, Dak Nong is a remote province in Vietnam, and the hospital can only recruit doctors who practice general medicine. Consequently, health workers at the provincial hospital are not confident in treating premature newborns. Similarly, the health workers at the district level hospitals refer any sick newborn babies because of limited confidence in managing neonatal complications.

*“We are not very confident in newborn diseases, especially the early premature newborns. For those cases, we refer to Ho Chi Minh city”* (Health worker, provincial level, Vietnam)*.*

*“We do not treat any neonatal disease in this hospital. All cases are referred”* (Health worker, district level, Vietnam).

*“Rural areas lack sufficient qualified medical staff to provide services, so these guidelines were not fully implemented. For example, according to the guideline on infant feeding, mothers should start to breastfeed newborns 1 hour after giving birth. However, the actual rate is merely 41 %”* (Key informant, national level, China).

*“China has developed large-scale neonatal resuscitation training to address the problem of neonatal asphyxia, but the training effect is barely satisfactory. As the training is provided from one level to the next lower level, there is some information loss during the process. Insufficient supervision following the training is another problem”* (Key informant, national level, China).

##### Shortage of funding

In Vietnam, respondents reported that there is no specific budget for neonatal health care, but estimates have shown that funding only meets about 45 % of the actual demand. In China, this was not reported as an issue.

“*Neonatal health is now the main target for MCH program. However, the budget for neonatal health care has not met the demand. In the short term, we will focus on training and simple interventions on essential neonatal care. In the meantime, we need to call for further international support*” (Key informant, national level, Vietnam).

##### Cultural: reconciliation with patients’ practices and views

There are some cultural practices within the community that affect implementation of the guidelines. In Vietnam, there are some traditional postnatal practices that are not consistent with the guidelines. For example, women from some ethnic minority groups discard colostrum or feed the baby with honey. Health workers find it difficult to influence these practices. In China, the “*Zuo Yuezi*” or “confinement following delivery” prohibits women and babies leaving the home and receiving visitors for 1 month following birth.

“*Ethnic minority people have their own cultural practices. Although we instruct about breastfeeding, they sometimes feed the newborns with honey. They think that honey is good for babies’ digestion. These practices are more popular after the women and their babies are discharged from the health facilities”* (Health worker, district level, Vietnam).

##### Involving the community in provision of care

In Vietnam, there were several examples of community actions that currently support neonatal health. The Women’s Unions (civil society organisation funded by the government which promotes women’s activities) are involved in counselling women on antenatal care, postnatal care and breastfeeding and organising health promotion activities on the radio. The Commune People’s Committees provide financial and logistical support to the commune health stations to deliver maternal and child health communication activities. Local authorities and community leaders are involved in selecting women to participate in the Village Based Ethnic Minority Midwives Initiative, thus facilitating their status and acceptance by communities. Village health workers and ethnic midwives act as bridges between the formal health system and communities.

However, in China, apart from activities by the village doctors, we did not identify any community actions related to neonatal health that were organised by community organisations.

##### Management support: Enabling the use of guidelines

The findings suggest that managers can facilitate the use of guidelines if and when they can provide the necessary resources including staffing, equipment and supplies as well as involving the community.

### Other management support

#### Supervision

In Vietnam, routine supervision covers the implementation of the guidelines. However, health workers who do not follow the guidelines are not held accountable. If health workers have not been trained in use of guidelines, then they will not follow the guidelines.

In China, the respondents reported that implementation of guidelines is not promoted through the routine supervision mechanisms. Managers do not monitor how the guidelines are implemented, and they are not reflected in the supervision process and documentation. There is no accountability of health workers to follow the guidelines.

*“China’s NH guidelines face the issue of poor enforcement. This is partially due to lack of powerful supervision and accountability mechanism during execution of guidelines. For instance, exclusive breastfeeding is mentioned in all relevant guidelines, but the implementation status is still very poor”* (Key informant, national level, China).

#### Lack of coordination for NH at national level

In Vietnam, there are several issues with the coordination of neonatal health care services at the national level. The number of MoH neonatal health focal persons is insufficient to cover all neonatal care activities. There is inadequate coordination between the preventive health and treatment sub-sectors. There is no focal person for neonatal health at the Health Service Administration (which is responsible for organising, developing policy and carrying out supportive supervision) causing difficulties in conducting supportive supervision at health care facilities. The technical group at the national level for neonatal health was established but is not currently functioning.

#### Challenges with monitoring

Monitoring implementation of guidelines and their effects on service delivery and health outcomes is an important managerial function. In Vietnam, the Health Information System collects data on some neonatal health indicators at all levels in the health system. The Provincial Reproductive Health Centre and the MCH department of the MoH collect, analyse and publish some neonatal health data. In China, the routine data reporting system which operates at the village, township, county, province and national levels of the health system collects data on many neonatal health indicators such as neonatal deaths, stillbirths, low birth weight and breastfeeding.

However, respondents reported several challenges with monitoring neonatal health in China and Vietnam. They explained that the accuracy of the data may be compromised for several reasons: there are too many indicators, some are reported as estimates at facility level, and there is under reporting of neonatal deaths by families and health workers. In Vietnam issues include: limited capacity of staff to manage and analyse data; poor financing of the system resulting in inability to check the data in the routine reports; lack of computers and software to carry out analysis. In China, not all information is reported electronically, and entering data from paper forms is time consuming and can result in errors. In China there is over reporting of some indicators such as breastfeeding and postnatal visits and data from migrant population is not captured. In addition, the data is often not disaggregated by gender or socio economic status.

Respondents in China and Vietnam reported that there is limited use of the data for informing decisions about delivery of NH services. In Vietnam, it appears that there is limited understanding of how to use the routine data to inform planning and resource allocation at the local and national levels. The MoH prefers to use survey data such as Multiple Indicator Cluster Surveys and Demographic Health Surveys to inform policy and resource allocation, as it is seen as being more reliable. In China, data are used for comparison between provinces, programme evaluation, and sometimes for international comparison. There is no evidence that local health authorities and health facilities use the data to improve NH services.

#### Managers’ room for manoeuvre to promote use of guidelines

In Vietnam, managers at the provincial level have more room for manoeuvre due to their professional position, responsibilities and knowledge, as well as the Provincial Hospital being financially autonomous. They have the authority to decide which services to provide. Managers at the district and sub-district level institutions appear to have little autonomy and are unable to decide which services to provide.

In China, managers at county and township levels do not appear to have full control over their budgets or human resources. This means they do not get sufficient quantity and quality of staff to provide required services. Recruitment of staff is done on request by the facility managers to the county health bureau and bureau of human resources and social security. All health facilities can decide training related issues, including training content and selection of participants. Township health centre and village health centre staff cannot decide the content of programmes such as postnatal care as this is designed by national level, but they can decide when and how to implement the programmes.

## Discussion

Using the behaviour change framework by Cabana et al [6], this study explored the use of neonatal health guidelines including barriers, and the role of management in supporting effective usage in China and Vietnam. It has confirmed that for effective use of guidelines, all three main areas-knowledge of guidelines, attitudes towards guidelines and factors affecting behaviour-need to be investigated for potential barriers, as well as facilitating factors [6]. We found that Cabana’s model was useful once adapted to include management support. We found that guidelines are not readily available at county, township and village levels in China, whereas, in Vietnam, guidelines are available at facility level, are accepted and are being used. Improvements in implementation could be made in both settings. Several factors influencing the use of the guidelines common to both settings included: lack of equipment and supplies; shortage of staff especially with neonatal health care experience; shortage of funding specifically for NH; and guidelines not in line with patient practices and views. There were several issues with guidelines use specific to China and these included: issues with dissemination of guidelines to the county and lower levels of the health system; lack of agreement with the guidelines; and preferring to provide care according to habit and routine. There was limited community engagement in neonatal health services in China, whereas in Vietnam, there were examples of how community members were actively involved in decision making and provision of services. Health facility managers have an important role to play in supporting the use of neonatal health guidelines in their settings through: ensuring that guidelines are available; allocating appropriate resources; supporting and monitoring staff in their use; and building relationships with local communities to engage them in health services.

The following section explores four areas emerging from these findings: importance of effective dissemination strategies, staffing and other resources being critical for guidelines adherence, community engagement in neonatal health, and the role of managers in supporting guideline use.

### Effective dissemination strategies are essential for guideline use

Dissemination of guidelines is easier when there is one set of nationally recognised guidelines, as is the case in Vietnam. There appears to be a clear strategy for dissemination being implemented which includes training on guidelines use which has resulted in guidelines being available and training received even in the remote areas. However, effectiveness of the training in developing intended skills, was questionable. In China, there is little evidence of the existence of a strategy for dissemination of guidelines, and training is ad hoc and does not appear to be related to any guidelines. This has resulted in guidelines not being readily available, a lack of awareness about the guidelines among NH care providers, and a vacuum of standardised and evidence based care. Providers therefore found other options: they either referred to textbooks to guide their care, used guidelines they found on the internet, continued their previous practice, or followed the practice of other colleagues. Using other guidelines than the “official” ones undermines one of its most important values which is to standardise evidence-based clinical practice. Part of the role of managers at the different levels of the health care system, is to ensure that guidelines are available to the staff and that staff are aware of the content [31]. Other studies have shown that multiple strategies for dissemination that include initial and ongoing training, face to face instruction, monitoring and feedback involving key stakeholders such as service providers, managers and users can be effective in improving use of guidelines [32–35]. Further research that explores the specific barriers to dissemination of guidelines in China, synthesises learning from other contexts where guideline dissemination has been successful, designs strategies for the China context, and evaluates them in terms of effectiveness, feasibility and acceptability.

### Staffing and other resources are critical for guidelines adherence

There are also problems with resources, including adequate numbers and appropriately skilled staff, and limited drugs and equipment, which influence how guidelines are followed in China and Vietnam. It is important that guidelines do acknowledge these challenges and where possible provide alternatives for situations where resources are limited. A review of hypertension management guidelines in China found that the actual implementation of the guidelines was not addressed and recommended that defining facilitators and barriers for adoption of guidelines should be integrated early in the guideline development process [36]. This principle should be applied also in the adoption of NH guidelines.

Motivation of staff to adhere to guidelines can be promoted through the provision of the necessary drugs, supplies and equipment as well as ensuring they have the competencies and confidence to implement them. Lack of these essential requisites can affect health workers’ motivation to provide services [37]. In addition, service users can bypass facilities where there is a lack of resources, and this makes it difficult for staff to acquire the necessary clinical experience to maintain knowledge and skills [38]. Recognition by managers and communities and feeling of achievement also influence staff motivation, adherence to guidelines and performance [37].

As indicated in this study and supported by others, an important role of managers is to ensure that their staff have the skills and knowledge, and resources to perform their work [39]. This can be assessed through human resource management tools such as appraisal and supervision, as well as meetings with staff, observations of the facility and through feedback from service users. It is important for managers to select the most appropriate human resource management tools for the context [37]. Allocation of existing resources and leverage for additional resources to support the use of guidelines, whether this is for extra staff, training or new equipment, is also an essential function of the manager.

Further research should explore options to improve the skills and knowledge of health workers to support the implementation of guidelines through blended learning approaches such as: focused competency based workshops; e-learning tools including webcasts, simulation training, social media, open online courses which can also support peer to peer learning, joint problem solving and reflective practice; and mentorship.

### Community engagement is needed to complement the use of NH guidelines

Improvements can be made to the supply side of neonatal health care, and guidelines would play an important part in ensuring quality. However, at the same time complementary work is needed on the demand side. Interventions to promote appropriate health seeking behaviour amongst communities, can influence and push health care providers to adopt and adhere to existing guidelines. This can be particularly important where local customs can either not support or act as a barrier to the use of guidelines e.g. the local custom of discarding colostrum, acts a barrier to guidelines promoting early breastfeeding. The challenge for managers would be to ensure that both supply and demand are covered with effective and coherent interventions to promote the use of guidelines.

Models of community engagement that support provision of care and increase demand and complement service delivery are available (e.g. use of women’s groups, village health workers). These community engagement activities may be particularly important in less developed locations where people are further from formal services during the critical neonatal period [40]. In Vietnam, there is already a strong foundation on the demand side, with active women’s unions, the Commune People’s Committee and village health workers and ethnic midwives. Other studies in Vietnam and elsewhere have highlighted the importance of community engagement in improving maternal and neonatal health [15, 41–43].

In contrast, in China, apart from the village doctor who is the link between communities and the formal health system, there was little engagement with communities in the study sites. Women’s Committees are more engaged with family planning rather than neonatal health. There are few examples of community engagement in neonatal health care in China in the literature. However, these provide positive examples. In rural Tibet, where many women had a home delivery without skilled birth attendance, an outreach programme provided maternal-newborn health education, skills training, and resources to the home including basic neonatal resuscitation and cord care [44]. The Women’s Reproductive Health and Development Program in Yunnan Province, promoted community engagement in women’s reproductive and family health [45]. Further research is needed to design, implement and evaluate community engagement models that increase demand for and use of neonatal healthcare services.

### Managers have a vital role to play in supporting guidelines use

As discussed above, managers have a vital role to play in supporting guidelines use. It is therefore critical that they are committed and perceive the guidelines as a valuable way of strengthening the performance of their organisation [46]. Monitoring how guidelines are used, and their effects on service delivery and health outcomes is an important function of managers. Monitoring progress with guideline use, audit of practice and provision of feedback were seen as enabling factors for the implementation of clinical guidelines in Mongolia [35]. The health information system and other sources of data such as surveys, can be used to help monitor and evaluate guideline use, and inform decisions about service delivery. However, the use of information systems for monitoring and evaluation of guidelines use is not evident from the study in China or Vietnam, particularly at local levels. There is a need to build capacity in using data from information systems and surveys for problem solving and decision making and to embed this approach in the way managers work. Equally essential, is feeding back this information to the providers and taking action accordingly. Understanding the challenges and facilitators of using the guidelines, and identifying ways to resolve problems can be gained through observation and dialogue with providers. The next phase of this research would test a management strengthening intervention where managers develop, implement, monitor and evaluate interventions to support better implementation of guidelines.

### Limitations of the study

This study addresses an important but relatively neglected area in neonatal health. This was a small scale preliminary study designed to be carried out rapidly to provide a snapshot of NH guideline use in one location in 2 countries. It was a qualitative study that captured reported health worker behaviour about use of guidelines, and did not use observation of actual health worker behaviour, or routine data from registers or patient notes about clinical practice. We did not aim to produce generalizable findings, but to highlight the key challenges and opportunities surrounding NH guidelines use that can feed into a larger implementation research study, and contribute to our understanding of how best to support implementation of guidelines as a means to improve NH. Confirmation of key themes across the two contexts and the triangulation of perspectives, means that the results are likely to have a wider resonance and applicability. It is possible that participants may have felt they should report positive behaviour and attitude towards the guidelines; we aimed to address this through developing trusting relationships with participants and appropriate in-depth probing.

## Conclusions

Availability of guidelines at the facility level is an important factor influencing the adoption of guidelines in the study sites in China. In Vietnam, guidelines are available at facility level, are accepted and are being used. However, improvements in implementation could be made. The study showed that problems with use of guidelines focus around: availability and capacity of human resources; working environment; local customs; weak supervision and monitoring; and poor demand for care. There is need to engage managers to support implementation. Management systems that provide the necessary resources, competent staff, monitoring, regulatory and incentive frameworks as well as community engagement are needed to promote the adoption of and adherence to guidelines. Further research areas include: models of dissemination of guidelines; use of innovative training and mentoring approaches to improve health worker skills and knowledge to support the implementation of guidelines; implementation of community engagement models; and how best to strengthen local level management so that they tailor interventions to support guideline use to their specific context. This will ensure that proven interventions to address NH problems are used, and that countries move closer to achieving the new SDG target.

## Additional file

Additional file 1: Topic guides used in the semi-structured interviews and focus group discussions. (DOCX 28 kb)

## Abbreviations

FGD: Focus group discussion
MCH: Maternal and child health
MoH: Minstry of health
NGRHS: National guidelines for reproductive health care services
NH: Neonatal health
NMR: Neonatal mortality rate
SDG: Sustainable development goal
SSI: Semi-structured Interviews
