# Supplementary material for: Using guidelines to improve neonatal health in China and Vietnam: a qualitative study
Source: BMC Health Serv Res. 2016 Nov 11;16:647. doi: 10.1186/s12913-016-1900-x (PMC5106829; doi:10.1186/s12913-016-1900-x)
Supplement: Additional file 1: — Topic guides used in the semi-structured interviews and focus group discussions. (DOCX 28 kb) [file 12913_2016_1900_MOESM1_ESM.docx]

**Topic Guides**

1. **Key informant interview: national level policy makers**
2. **Key informant interview: regional / provincial level policy makers and senior health service officials**
3. **Key informant interview: district / county level managers**
4. **Key informant interview: district / county level health workers**
5. **Key informant interview: community representatives**
6. **Focus group discussion: women**
7. **Focus group discussion: community members**
8. **Key informant interview: national level policy makers**

**Topic guide**

**We would like** to talk with you about your views and experiences of neonatal health and health care in the country. So the neonate relates to the newborn baby from birth until 28 days old.

*Neonatal health situation in remote rural areas of China / Vietnam*

1. Tell me about the neonatal health situation in remote rural areas of China / Vietnam
   - What is the neonatal mortality rate? What are the causes of deaths? Where is mortality particularly bad?
   - What are the underlying factors for high neonatal mortality?
   - What about morbidity in neonates? What are the key causes of morbidity?

*Key neonatal health interventions and guidelines*

1. What are the key neonatal health care interventions that are being implemented in rural China / Vietnam?
   - Who implements them? Since when?
   - What are the effects?
2. How are the interventions financed?
   - Who pays for infrastructure? Where are the records for this held?
   - Who pays for salaries? Where are the records for this held?
   - Who pays for medicines and supplies? Where are the records for this held?
   - Who pays for other operating costs (facility and outreach)? Where are the records for this held?
   - What is the policy on user payments for delivery and neonatal services?
     1. Who pays, and for which services?
     2. Are payments out of pocket or covered by insurance? If the latter, what proportion of clients and costs are covered?
     3. At which level is information on user payments held?
3. What guidelines are available to help health care providers deliver this care?
   - Where are they available?
   - When were they last updated?
   - What do you think of these guidelines?
   - Are there any issues with availability?

*Challenges in implementation of guidelines*

1. What are the challenges in implementing these guidelines in rural areas? (Probes: number of staff; skills and knowledge of staff; lack of drugs and materials; lack of transport etc.)
   - Describe these challenges in detail; are these challenges specific to neonatal health guidelines or to all guidelines?
2. What are the causes of these challenges?
3. What are the solutions to these challenges?

*Methods and challenges of monitoring neonatal health in rural/remote areas*

1. What are the methods of monitoring neonatal health in rural areas?
   - Probe for different types of methods such as surveys, routine collection of data – HMIS, perinatal death audits
   - Do these methods only capture what happens in facilities? What about what happens in the community?
2. What are the challenges in monitoring neonatal health?
   - Unreliable data? What are the causes?
   - Stigma and sensitivity of reporting neonatal deaths by families?
   - Stigma and sensitivity of reporting neonatal deaths by health care providers?
   - Anything else?

*Decision making space of district / county level managers*

1. What is the level of freedom/autonomy of district or county level managers with the way that they implement the guidelines?
2. What authority do district or county managers have to decide on use of existing or additional resources (e.g. staffing, transport, equipment, drugs) more successfully implement the guidelines?

*Policy space for introducing a problem solving intervention*

We have been talking about challenges of using the guideline [refer to specific examples discussed]. How are these sorts of problems addressed at district/county level?

1. Are there any programmes/projects that use a participatory problem solving approach?
   - How participatory are they?
   - How effective are they for problem solving?
   - Is this an approach that could be used more?

*Closure*

1. Is there anything else you would like to add?
2. **Key informant interview: regional / provincial level policy makers and senior health service officials**

**Topic guide**

**We would like** to talk with you about your views and experiences of neonatal health and health care in the country. So the neonate relates to the newborn baby from birth until 28 days old.

*Neonatal health situation in remote rural areas of your region / province*

1. Tell me about the neonatal health situation in remote rural areas of China / Vietnam
   - What is the neonatal mortality rate? What are the causes of deaths? Where is mortality particularly bad?
   - What are the underlying factors for high neonatal mortality?
   - What about morbidity in neonates? What are the key causes of morbidity?

*Key neonatal health interventions and guidelines*

1. What are the key neonatal health care interventions that are being implemented this region / province?
   - Who implements them? Since when?
   - What are the effects?
2. What guidelines are available here to help health care providers deliver this care?
   - Where are they available?
   - When were they last updated?
   - What do you think of these guidelines?
   - Are there any issues with availability?

*Challenges in implementation of guidelines*

1. What are the challenges in implementing these guidelines? (Probe: number of staff; skills and knowledge of staff; lack of drugs and materials; lack of transport etc.)
   - Describe these challenges in detail; are these challenges specific to neonatal health guidelines or to all guidelines?
2. What are the causes of these challenges?
3. What are the solutions to these challenges?

*Methods and challenges of monitoring neonatal health in rural areas*

1. What are the methods of monitoring neonatal health in your region / province?
   - Probe for different types of methods such as surveys, routine collection of data – HMIS, perinatal death audits
   - Do these methods only capture what happens in facilities? What about what happens in the community?
2. What are the challenges in monitoring neonatal health?
   - Unreliable data? What are the causes?
   - Stigma and sensitivity of reporting neonatal deaths by families?
   - Stigma and sensitivity of reporting neonatal deaths by health care providers?
   - Anything else

*Decision making space of district / county level managers*

1. What is the level of freedom/autonomy of district or county level managers with the way that they implement the guidelines?
2. What authority do district or county managers have to decide on use of existing or additional resources (e.g. staffing, transport, equipment, drugs) more successfully implement the guidelines?

*Policy space for introducing a problem solving intervention*

We have been talking about challenges of using the guideline [refer to specific examples discussed]. How are these sorts of problems addressed at district/county level?

Are there any programmes/projects that use a participatory problem solving approach?

- - How participatory are they?
  - How effective are they for problem solving?
  - Is this an approach that could be used more?

*Financing maternal and newborn health services*

1. How are maternal and newborn health services financed in the study areas?
   - What are the channels?
   - Which costs are borne by patients?
   - Are all patients costs paid out of pocket or do insurance schemes cover neonatal costs? If so, please describe how these work: who is a member, how are their premia paid; what costs are covered; and how well do they provide financial protection?
2. Which costs are paid centrally [insert relevant body e.g. MoH] and how available is this information for the study areas?
   - Probe for: staff salaries for each district and facility; running costs; drugs and supplies
3. What financial records are held at facility level?
   - If we are trying to cost NH interventions, would they be available and sufficiently accurate?
   - How is the financial information reported from the facility level to your level?
4. Is there existing information on patients’ payments for these services (within facility costs and external ones, e.g. for transport, drugs and food)?

*Closure*

1. Is there anything else you would like to add?
2. **Key informant interview: district / county level managers**

**Topic guide**

**We would like** to talk with you about your views and experiences of neonatal health and health care in the country. So the neonate relates to the newborn baby from birth until 28 days old.

*Neonatal health situation in your district / county?*

1. Tell me about the neonatal health situation in your district / county?
   - What is the neonatal mortality rate? What are the causes of deaths? Where is mortality particularly bad?
   - What are the underlying factors for high neonatal mortality?
   - What about morbidity in neonates? What are the key causes of morbidity?

*Key neonatal health guidelines*

1. What guidelines are available to help you deliver neonatal health care?
   - Where are they kept? Who developed them? Where did they come from? When did you receive them? How old are they? Can you show us a copy of the guidelines?
   - What do you think of these guidelines? Do they help health workers carry out their work? How?
   - Are there any issues with availability?

*Challenges in implementation of guidelines*

1. What are the challenges in implementing these guidelines? (Probe: number of staff; skills and knowledge of staff; lack of drugs and materials; lack of transport etc.)
   - Describe these challenges in detail
2. What are the causes of these challenges?
3. What are the solutions to these challenges?

*Methods and challenges of monitoring neonatal health in rural areas*

1. What are the methods of monitoring neonatal health in your region / province?
   - Probe for different types of methods such as surveys, routine collection of data – HMIS, perinatal death audits
   - Who collects the data?
   - Do these methods only capture what happens in facilities? What about what happens in the community?
2. What are the challenges in monitoring neonatal health?
   - Unreliable data? What are the causes?
   - Stigma and sensitivity of reporting neonatal deaths by families?
   - Stigma and sensitivity of reporting neonatal deaths by health care providers?
   - Anything else?

*Existing community engagement activities to support neonatal health in this district / county*

1. What neonatal health activities are happening within the community in this district/ county? Probe with some examples e.g. breastfeeding groups; mother’s groups;
   - Describe in detail
   - Who organised them? How do they collaborate with the health system? How are these activities funded?
   - What do you think of these activities?
   - What are the effects of the activities?
   - What are the challenges of implementing these activities?
   - How could these activities be improved?
2. Do you have any documents that describe neonatal health and community engagement activities? Please can you show these documents?
3. What other community activities to improve neonatal health (including demand for services) should be implemented?
   - Why these activities?
   - How should they be implemented? (Who? Collaborations? Funding?)

*Decision making space of district / county level managers*

1. What is the level of freedom/autonomy of district or county level managers with the way that they implement the guidelines?
2. What authority do district or county managers have to decide on use of existing or additional resources (e.g. staffing, transport, equipment, drugs) more successfully implement the guidelines?

*Financing maternal and newborn health services*

1. How are maternal and newborn health services financed in the study areas?
   - What are the channels?
   - Which costs are borne by patients?
   - Are all patients costs paid out of pocket or do insurance schemes cover neonatal costs? If so, please describe how these work: who is a member, how are their premia paid; what costs are covered; and how well do they provide financial protection?
2. Which costs are paid centrally and how available is this information for the study areas?
   - Probe for: staff salaries for each district and facility; running costs; drugs and supplies
3. What financial records are held at facility level?
   - If we are trying to cost NH interventions, would they be available and sufficiently accurate?
   - How is the financial information reported from the facility level to your level?
4. Is there existing information on patients’ payments for these services (within facility costs and external ones, e.g. for transport, drugs and food)?

*Feasibility and acceptability of using a participatory problem solving approach*

We have been talking about challenges of using the guideline [refer to specific examples discussed]. How are these sorts of problems addressed at district/county level?

1. Are there any programmes/projects that use a participatory problem solving approach?
   - How participatory are they?
   - How effective are they for problem solving? Is this an approach that could be used more?

*Closure*

1. Is there anything else you would like to add?
2. **Key informant interview: district / county level health workers**

**We would like** to talk with you about your views and experiences of neonatal health and health care in the country. So the neonate relates to the newborn baby from birth until 28 days old.

*Role in neonatal health care*

1. What is your role in neonatal health care?
   - What do you do? Please describe
   - How long have you been doing this?
   - Who manages you? Who supervises you? How do they do this? How often?

*Neonatal health situation in county / district*

1. Tell me about the neonatal health situation in your county / district
   - What is the neonatal mortality rate? What are the causes of deaths? Where is mortality particularly bad?
   - What are the underlying factors for high neonatal mortality?
   - What about morbidity in neonates? What are the key causes of morbidity?

*Key neonatal health guidelines*

1. What guidelines are available to help you deliver neonatal health care?
   - Where are they kept? Who developed them? Where did they come from? When did you receive them? How old are they? Can you show us a copy of the guidelines?
   - What do you think of these guidelines? Do they help you to carry out your work? How?
   - Are there any issues with availability?

*Challenges in implementation of guidelines*

1. What are the challenges in implementing these guidelines in rural areas? (Probe: number of staff; skills and knowledge of staff; lack of drugs and materials; lack of transport etc)
   - Describe these challenges in detail
2. What are the causes of these challenges?
3. What are the solutions to these challenges?

*Methods and challenges of monitoring neonatal health in rural areas*

1. What are the methods of monitoring neonatal health in your county / district?
   - Probe for different types of methods such as surveys, routine collection of data – HMIS, perinatal death audits
   - Who collects the data?
   - Do these methods only capture what happens in facilities? What about what happens in the community?
2. What are the challenges in monitoring neonatal health?
   - Unreliable data? What are the causes?
   - Stigma and sensitivity of reporting neonatal deaths by families?
   - Stigma and sensitivity of reporting neonatal deaths by health care providers?
   - Anything else?

*Existing community engagement activities to support neonatal health in this district / county*

1. What neonatal health activities are happening within the community in this district/ county? Probe with some examples e.g. breastfeeding groups; mother’s groups;
   - Describe in detail
   - Who organised them? How do they collaborate with the health system/health staff? How are these activities funded?
   - What are the effects of the activities?
   - What are the challenges of implementing these activities?
   - How could these activities be improved?
2. Do you have any documents that describe neonatal health and community engagement activities? Please can you show these documents?
3. What other community activities to improve neonatal health (including demand for services) should be implemented?
   - Why these activities?
   - How should they be implemented? (Who? Collaborations? Funding?)

*Closure*

1. Is there anything else you would like to add?

**5. Key informant interview: community representatives**

We want to find out about your views of health care services for babies aged under 1 month old (neonates).

*Neonatal health situation in county / district*

1. Tell me about the neonatal health situation in your county / district
   - Is neonatal death a serious problem in this area? What are the causes of deaths? Where is mortality particularly bad?
   - What are the underlying factors for high neonatal mortality?
   - What about illness in neonates? What are the key causes of illness?
   - How are neonatal deaths and illnesses reported? Is the reporting accurate? Are there any problems with this?

*Existing community engagement activities to support neonatal health in this district / county*

1. What neonatal health activities are happening within the community in this district/ county? Probe with some examples e.g. breastfeeding groups; mother’s groups;
   - Describe in detail
   - Who organised them? How do they collaborate with the health system and health workers? How are these activities funded?
   - What do you think of these activities?
   - What are the effects of the activities?
   - What are the challenges of implementing these activities?
   - How could these activities be improved?
2. What other community activities to improve neonatal health (including demand for services) should be implemented?
   - Why these activities?
   - How should they be implemented? (Who? Collaborations? Funding?)

*Closure*

1. Is there anything else you would like to add?
2. **Focus group discussion: women**

We want to find out about your experiences of using health care services for your baby, at birth and when he or she was less than one month old.

*Experiences of neonatal health care services*

1. Please can you tell us about when you had the baby?
   - Where was the baby born? At home or in facility?
   - Who delivered the baby?
   - What happened when the baby was born? (Probe: early breastfeeding, keeping baby warm, skin to skin contact with mother, vaccination, vitamin K prophylaxis, counselling before discharge about feeding, vaccinations, baby care, danger signs etc)
2. Please can you tell me about the last time you used health care services or were visited by a health care worker for your baby when he / she was less than one month old.

- Why did you visit the health facility with your baby? Why did the health worker visit?
- What services were provided? Explain what happened when you received the services / visit?
- Who provided them?
- Where did you go?

*Perceptions of neonatal health care services*

1. What do you think of the care that your baby received?
2. What do you think about the health care provider who gave this care?
3. What were the good points about when you went for these services?
4. What are the bad points about when you went for these services?

*Ways to improve services*

1. What areas need to be improved?
   - How can they be improved?
   - Why should they be improved?

*Existing community engagement activities to support neonatal health in this district / county*

1. What neonatal health activities are happening within the community in this district/ county? Probe with some examples e.g. breastfeeding groups; mother’s groups; women’s unions;
   - Describe in detail
   - What do you think of these activities?
   - How could these activities be improved?
2. What other community activities to improve neonatal health should be implemented?
   - Why these activities?
   - How should they be implemented? (Who? Collaborations? Funding?)

*Reporting neonatal mortality and morbidity*

1. How are deaths and illnesses of young babies (aged under 1 month) reported? Is the reporting accurate? Are there any problems with this?

*Closure*

1. Is there anything you would like to add?
2. **Focus group discussion: community members**

We want to find out about your experiences of using health care services for the baby of one of your relatives, when he or she was less than one month old.

*Experiences of neonatal health care services*

1. Please can you tell us about when your family member had the baby?
   - Where was the baby born? At home or in facility?
   - Who delivered the baby?
   - What happened when the baby was born? (Probe: early breastfeeding, keeping baby warm, skin to skin contact with mother, vaccination, vitamin K prophylaxis, counselling before discharge about feeding, vaccinations, baby care, danger signs etc)
2. Please can you tell me about the last time you or your family used health care services or were visited by a health worker for your baby when he / she was less than one month old.

- Why did you visit the health facility with your baby? Why did the health worker visit?
- What services were provided? Explain what happened when you received the services / visit
- Who provided them?
- Where did you go?

*Perceptions of neonatal health care services*

1. What do you think of the care that your baby received?
2. What do you think about the health care provider who gave this care?
3. What were the good points about when you went for these services?
4. What are the bad points about when you went for these services?

*Ways to improve services*

1. What areas need to be improved?
   - How can they be improved?
   - Why should they be improved?

*Existing community engagement activities to support neonatal health in this district / county*

1. What neonatal health activities are happening within the community in this district/ county? Probe with some examples e.g. breastfeeding groups; mother’s groups; women’s unions
   - Describe in detail
   - What do you think of these activities?
   - How could these activities be improved?
2. What other community activities to improve neonatal health should be implemented?
   - Why these activities?
   - How should they be implemented? (Who? Collaborations? Funding?)

*Reporting neonatal mortality and morbidity*

1. How are deaths and illnesses of young babies (aged under 1 month) reported? Is the reporting accurate? Are there any problems with this?

*Closure*

1. Is there anything you would like to add?
